# Supplementary material for: Identification of photoperiod-induced specific miRNAs in the adrenal glands of Sunite sheep (Ovis aries)
Source: Front Vet Sci. 2022 Jul 22;9:888207. doi: 10.3389/fvets.2022.888207 (PMC9354845; doi:10.3389/fvets.2022.888207)
Supplement: Supplementary file 1 [file Data_Sheet_1.ZIP › Supplementary file/miRDeep2 file.zip!/miRDeep2 file/Know_Structure/SP42A1/oar-mir-376c.pdf]

miRBase precursor : oar-mir-376c
Total read count : 470
oar-miR-376c-5p read count : 149
oar-miR-376c-3p read count : 321
remaining reads : 0

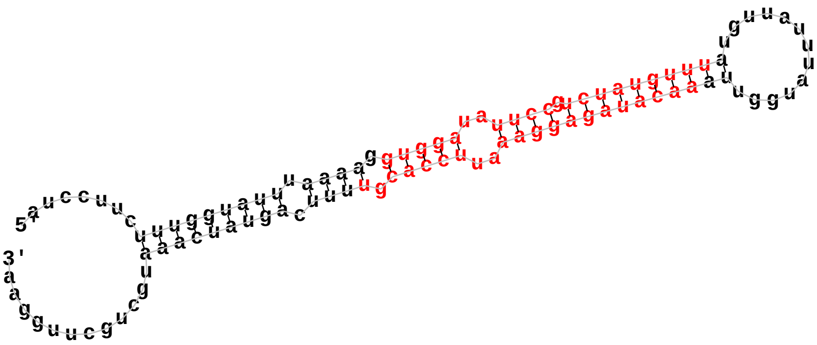

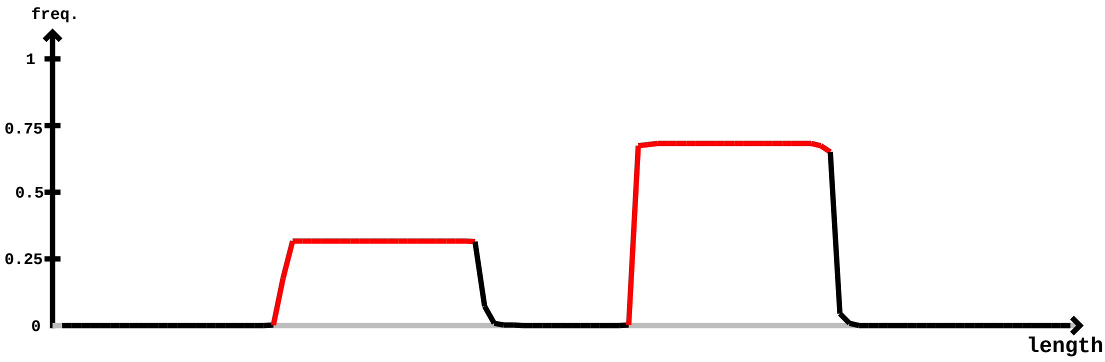


```
                                                        oar-miR-376c-3p
                          oar-miR-376c-5p
5'- auccuucuuugguauuuaaaagguggauauuccgucuauguuuauguuauuuaugguuaaacauagaggaaauuccacguuuucaguaucaaaugcugcuuggaa   -3'    exp
    .......(((((((((.((((.(((((..((((.((((((((((............))))))))))))))..))))).)))).))))))))).............     reads      mm        sample
    .....................gguggauauuccgucuauguuu..............................................................     1          0         seq
    ......................guggauauuccgucuauguuu..............................................................     65         0         seq
    ......................guggauauuccgucuauguuua.............................................................     17         0         seq
    .......................uggauauuccgucuauguu...............................................................     1          0         seq
    .......................uggauauuccgucuauguuu..............................................................     48         0         seq
    .......................uggauauuccgucuauguuua.............................................................     13         0         seq
    .......................uggauauuccgucuauguuuau............................................................     3          0         seq
    .......................uggauauuccgucuauguuuaugu..........................................................     1          0         seq
    ..........................................................aaacauagaggaaauuccac...........................     1          0         seq
    ...........................................................aacauagaggaaauuccac...........................     3          0         seq
    ...........................................................aacauagaggaaauuccacg..........................     11         0         seq
    ...........................................................aacauagaggaaauuccacgu.........................     285        0         seq
    ...........................................................aacauagaggaaauuccacguu........................     15         0         seq
    ...........................................................aacauagaggaaauuccacguuu.......................     2          0         seq
    ............................................................acauagaggaaauuccacguu........................     2          0         seq
    .............................................................cauagaggaaauuccacguuu.......................     2          0         seq
```
